# Unlocking the connection between education, entrepreneurial mindset, and social values in entrepreneurial activity development

**DOI:** 10.1007/s11846-023-00629-w

**Published:** 2023-02-10

**Authors:** Lurdes D. Patrício, João J. Ferreira

**Affiliations:** 1 Escola Superior de Tecnologia e Gestão de Viseu &, CISED – Centre for Research in Digital Services, Universidade da Beira Interior & NECE – Research Unit, Covilhã, Portugal and Instituto Politécnico de Viseu, Viseu, Portugal; 2 Universidade da Beira Interior & NECE – Research Unit, Covilhã, Portugal & QUT Australian Centre for Entrepreneurship Research, Australia, 6200- 209 Polo, Covilhã, IV Portugal

**Keywords:** Entrepreneurship, Entrepreneurial University, Educational level, Entrepreneurial attitudes, Social values, Total early stage entrepreneurial activity, Graduate, GEM, fsQCA

## Abstract

The Entrepreneurial University constitutes a phenomenon that highlights the prominent roles played by academic organizations as aggregators of capabilities, enabling the establishing of bridges between innovation and Entrepreneurial Ecosystems. This research therefore sets out to analyze the relationship between the Total Early Stage Entrepreneurial Activities of individual graduates and their entrepreneurial attitudes and social values towards entrepreneurship. This applies data sourced from the Global Entrepreneurship Monitor report on innovation-driven countries. The main research findings stem from the regression models (Study 1) and fsQCA analysis (Study 2) returning evidence that the likelihood of adult graduates setting up firms or owning young companies rises whenever such individuals deem they hold the knowledge/skills required to start a business. The results also stress the importance of devoting high levels of media attention to entrepreneurship and fostering entrepreneurial cultures capable of fostering economic growth and prosperity. This research makes substantial theoretical contributions to the literature. Firstly, the findings reinforce the applicability and suitability of fsQCA analysis of Global Entrepreneurship Monitor data. Secondly, this study strengthens the credibility of the Institutional Theory and Theory of Planned Behavior theoretical frameworks, correspondingly lending support to the importance of institutional or organizational factors as determinants of entrepreneurship and the need to focus on the linkage between entrepreneurial attitudes, entrepreneurial intentions, and entrepreneurial behaviors.

## Introduction

When placing the university as a key factor of economic change, it becomes possible to establish a parallel between the profound shift in the university model and the four industrial revolutions (Kochetkov et al. 2017). In this context, the university emerges as one of the main pillars of an ecosystem (Guerrero et al. 2017), responding to emerging socio-economic challenges (Kirby 2006), contributing to economic development, innovation, and the competitiveness of companies, regions, and countries (Fernandes and Ferreira 2013; Huggins, Johnston, and Stride 2012). In this sense, universities act as innovation intermediaries in knowledge transfer processes of increasing complexity in keeping with the diversity of participating partners (e.g., companies, government, and societal actors (Feser 2022).

Furthermore, integrating into entrepreneurial ecosystems may influence legitimation processes in which assessing the factors ensuring the optimization of entrepreneurial inputs is an stage (Bouncken and Kraus 2022). This positioning is based on the combination of the university’s traditional missions, based on teaching (first mission), research (second mission), and the emergence of an entrepreneurial academic spirit (Etzkowitz 2003) that emphasises entrepreneurship (third mission) and the corresponding emergence of the entrepreneurial university (Rubens et al. 2017). The entrepreneurial university therefore emerges as a catalyst for regional economic and social development (Urbano and Guerrero 2013). In entrepreneurial economies that extend beyond the role of small businesses and their owners, we also have to consider an all-embracing socio-economic mindset that equates to the opportunities existing and does not only focus on the availability of resources (Guerrero et al. 2015).

The Triple Helix concept usually serves to aggregate the Government-University-Industry cooperation perceived as crucial to economic progress with universities representing the enablers of qualified and specialized labor (Chen et al. 2016) through an appropriate alignment of university research and education and training priorities with the goals of the organizations active in the region (Hewitt-Dundas 2012). An entrepreneurial university thus nurtures a range of employability alternatives in their graduates, such as being self-employed, academic entrepreneurs or intrapreneurs (Guerrero et al. 2020).

It is important to bear in mind that new business creation is essential to generating employment, with education and training classing as core factors for fostering entrepreneurship, endowing graduate entrepreneurs with the rational capacity to develop initiatives with greater capacity for survival and growth (Coduras et al. 2008). The dynamics of entrepreneurial universities reflect the preponderant role that academic organizations play in shaping the aggregate capabilities (Audretsch et al. 2019) prevailing and building bridges between sources of innovation and entrepreneurial ecosystems (Autio et al. 2014).

Recognizing the paramount importance of universities as facilitators of contemporary knowledge and as the educators of potential entrepreneurs for society, exploring the interactions between university and entrepreneurship holds particular relevance (Coduras et al. 2008). Thus, considering how the interactions between university experiences and entrepreneurial activities open up vast and exciting research possibilities, there is clear interest in contributing by exploring the complexity of entrepreneurship based on Global Entrepreneurship Monitor (GEM) data (Coduras et al. 2016).

Through integrating the concepts described above, our research addresses the relevance of entrepreneurial universities in empowering potential entrepreneurs and reflects the need to further explore the complexity of entrepreneurship. The academic relevance of our research arises from the lack of studies establishing models that interrelate university support, attitudes, values and entrepreneurial activities – and, from the economic policy point of view - examining the relevance of promoting entrepreneurship within and from university contexts constitutes an essential facet.

In keeping with the literature and inspired by the gaps therein identified, our research analyzes the relationship between the Total Early Stage Entrepreneurial Activity (TEA) of graduates and the entrepreneurial attitudes and social values towards entrepreneurial activities. As previous GEM research has shown, taking into account the different types of economy is essential – factor-driven, efficiency-driven, and innovation-driven - based on the World Economic Forum’s classification (Coduras et al. 2016). In accordance with how relationships between entrepreneurship and economic development differ over the different phases of economic development, and also considering that innovation-driven countries rank highest for competitiveness according to the Global Competitiveness Report (Bosma et al. 2008), our study focused on innovation-driven countries in order to integrate the importance of the interactions of individuals with the environment within the scope of developing entrepreneurial activities. Our research deploys a combination of the quantitative approach - regression methodology (Study 1) with qualitative analysis – the fsQCA method (Study 2), thus enabling more detailed answers that explain the levels of entrepreneurial activities undertaken by individuals holding university qualifications with a more extensive explanation thereby feasible than those returned by the results of the regression models. The application of fsQCA serves to explore and exploit a new research framework and enrich our knowledge about the complexity of entrepreneurship by establishing different combinations of conditions that reflect the entrepreneurial attitudes and values prevailing in the countries studied (Coduras et al. 2016).

This study makes three fundamental contributions. Firstly, this contributes to recent work on the relationship between university support for entrepreneurship and the subsequent development of entrepreneurial activities, exploring the role of entrepreneurial education on successful entrepreneurial behaviors. The articulation of quantitative and qualitative methodologies also opens a range of promising possibilities. Should we be able to quantitatively assess the factors determining entrepreneurial activities, we will be able to qualitatively ascertain the eventual combinations between the factors under analysis and characterize their respective contexts. Secondly, this study strengthens the credibility of the Institutional Theory and Theory of Planned Behavior theoretical frameworks in lending support to the importance of institutional and organizational factors as determinants of entrepreneurship (Abreu et al. 2016) coupled with the linkage between entrepreneurial attitudes, entrepreneurial intentions and entrepreneurial behaviors (Ajzen 1991). In this sense, these theories are applicable to analysing the specific contributions of universities to the development of entrepreneurial activities by their graduates alongside ascertain the relevance of universities and account for the entrepreneurial performance prevailing in particular contexts. Thirdly, this reinforces the applicability and suitability of fsQCA analysis to GEM data. In contrast with previous approaches that focus on linear regression models, this study deploys fsQCA models to establish different combinations of the multiple and interrelated entrepreneurial conditions to generate the scope for defining the most effective and sustainable public policies in accordance with the characteristics of each country.

Following this introduction, this study has the following structure: the next section set out our review of the literature on the theme under analysis. We subsequently present the research data and methodology followed by the presentation of the results of Study 1 and Study 2. Finally, we discuss the findings and the conclusions.

## Theoretical background

The theoretical framework applied in this research derives both from institutional theory - grounded on institutional or organizational cultural, social, political and economic factors as the determinants of entrepreneurship (Abreu et al. 2016) – and from the theory of planned behavior – which draws attention to the interlinkage between entrepreneurial attitudes, entrepreneurial intentions and entrepreneurial behaviors on the grounds that becoming an entrepreneur represents consciously planned behavior (Ajzen 1991).

Drawing attention to the institutional or contextual factors - cultural, social, political, and economic - as the determinants of entrepreneurship, institutional theory stands out as one of the most appropriate theories for analysing the influence of environmental factors on entrepreneurship (Coduras et al. 2008). Organizations represent social and cultural systems that expect their members to engage in that deemed appropriate behavior with institutional theory returning an understanding of innovation that always considers the influence of this institutional context (Hinings et al. 2018). In addition, we may conceive of change as an outcome of social, organizational and individual structures, activities and actions (Scott 2014). The conceptual framework of institutional theory serves to approach the transition of universities from conservative institutions to leading entrepreneurial universities (Yoshioka-Kobayashi 2019), in compliance with the third mission (Kitagawa et al. 2016) and the relationship between university support for entrepreneurship and the actual development of entrepreneurial activities (Coduras et al. 2008).

Initially presented by Ajzen (1991), the theory of planned behavior posits that reasoning, control and planning underpin human social behavior. Hence, this foresees certain consequences arising from given behaviors (Ajzen and Fishbein 2000). Applied to predict many types of human behaviors (e.g., electoral choices, intentions to stop smoking, etcetera), the theory provides a meaningful framework for analysing the emergence of entrepreneurial behaviors (Fayolle et al. 2006). Accounting for actions in specific contexts (Ajzen 1991), and taking into account how the effects of entrepreneurial education take many years to bear fruit, isolating the role of entrepreneurial education within the scope of its capacity to nurture successful entrepreneurial behaviors is essential to ensuring the theoretical support of planned behavior theory (Fayolle et al. 2006). Furthermore, this educational exposure to entrepreneurship may or may not lead to later entrepreneurial activities. Despite the lack of general acceptance that entrepreneurial education generates some degree of preparedness for entrepreneurs – as is the case in the education of future practitioners such as doctors, lawyers, and engineers – it is difficult not to notice the benefits of entrepreneurial practices (Hindle 2007).

### Entrepreneurial ecosystems

The substantial transformations induced by digital technologies, the constantly changing demands of consumer as well as environmental uncertainties combine to create multiple challenges for companies that correspondingly need to display innovation to overcome the obstacles associated with disruptive business models (Stoiber et al. 2022). Establishing a social and economic environment for innovative and entrepreneurial endeavors requires the effective work of multiple and interconnected actors, including government entities, the private sector, society, universities and entrepreneurs themselves (Bouncken and Kraus 2022). Within the networks establishing between the multiple stakeholders shaping their configuration, evolution and outcomes, the entrepreneurial ecosystem emerges as a central topic on the agenda of researchers and political leaders (Fernandes and Ferreira 2022). Drawing on different perspectives and theories, the entrepreneurial ecosystem has become a theme of great interest in the academic community with the literature containing multiple studies defining the concept (Isenberg 2010, 2011; Stam 2015) alongside studies bringing together a set of definitions enabling the joint analysis of multiple perspectives on the concept (De Brito and Leitão 2021). Resulting from a biomimetic analogy, the entrepreneurial ecosystem is best described as a “*set of interdependent actors and factors coordinated in such a way that they enable productive entrepreneurship within a particular territory*” (Stam 2015, p.5). The entrepreneurial ecosystem sets the systemic conditions for interactions that influence the identification and commercialization of entrepreneurial opportunities (Audretsch et al. 2017). Centred on networks of entrepreneurs, leadership, finance, talent, knowledge and support services (Stam 2015), entrepreneurial ecosystems should foster favorable environments for innovation-based undertakings (Spigel 2017). In this scenario, innovation emerges out of the spatial concentration of principal and supporting companies that intentionally combine internal and external knowledge flows (De Brito and Leitão 2021). Adopting another perspective, Muldoon et al. (2022) explore the extended and blurred boundaries of entrepreneurial ecosystems, spanning the physical and cyber levels where technology has dissolved locational barriers and connected the different actors to enable the existence of social relationships that guarantee access to greater resources.

**Fig. 1:**
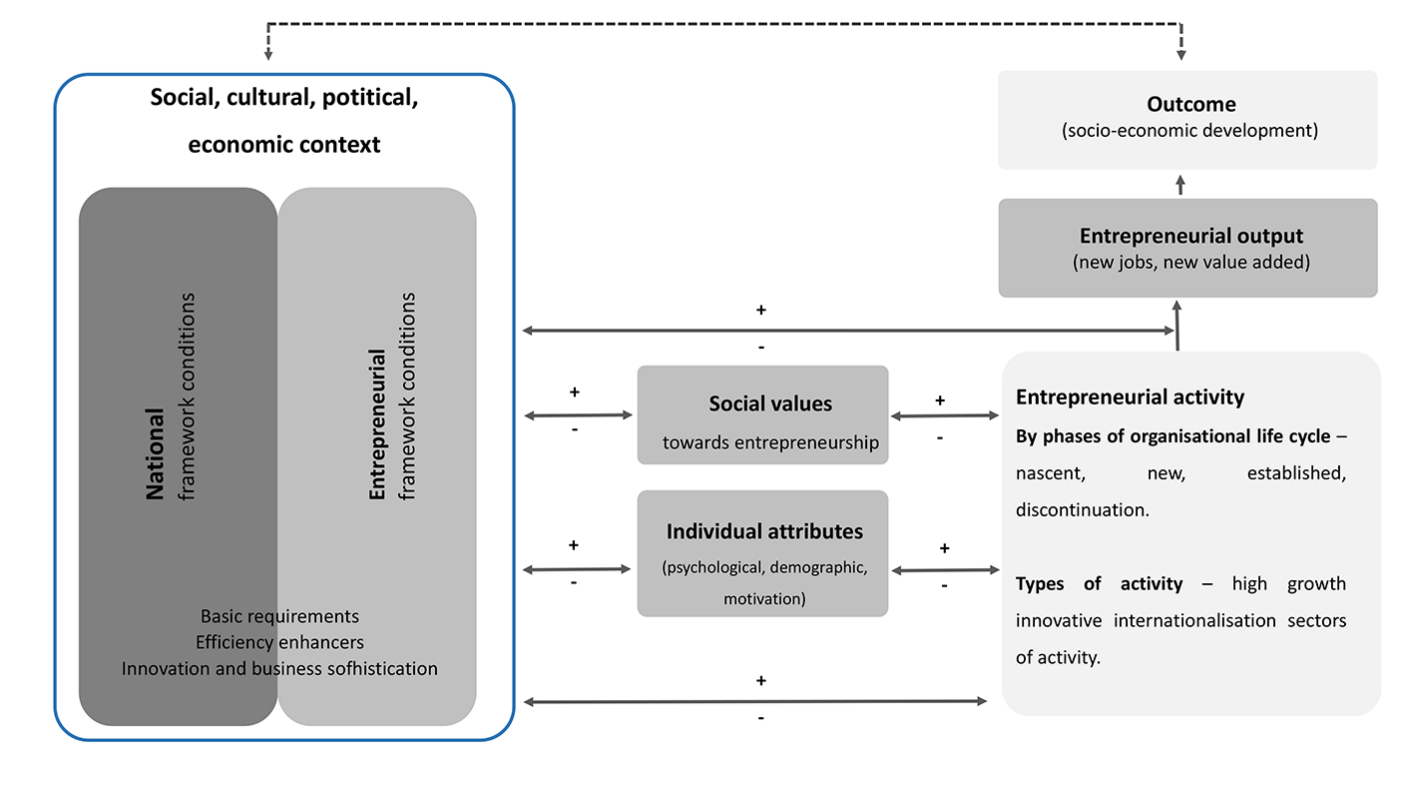
– GEM Conceptual Framework Source: GEM, 2017.

The GEM 2021 / 2022 Global Report (GEM 2022) describes and assesses entrepreneurial ecosystems according to nine factor framework - entrepreneurial finance, government policy, government entrepreneurial programs, entrepreneurial education, research and development transfers, commercial and professional infrastructures, ease of entry, physical infrastructures, and social and cultural norms towards entrepreneurship. The GEM theoretical framework (Fig. 1), resulting from the successive findings of the GEM scientific community, constitutes a suitable approach to examining the intermediate relationships between social values and attitudes and entrepreneurial activities.

The GEM evolution and transformation also take into consideration the different types of economy in keeping with the World Economic Forum’s classification – factor-driven, efficiency-driven, and innovation-driven (Coduras et al. 2016) to reflect the importance of the interactions of individuals within this environment to developing entrepreneurial activities (Bosma et al. 2008).

### Entrepreneurial universities and entrepreneurial cultures

As sources of knowledge, universities have to be based on an organizational culture that incorporates flexibility and is open to change, enabling the encouragement of entrepreneurship through providing more proactive and engaged responses to solving problems and generally improving society (Ferreira et al. 2018). Perceived as catalysts of regional and national economic and social development (Etzkowitz et al. 2000; Abreu et al. 2016), entrepreneurial universities fuel their surrounding entrepreneurial ecosystems (Audretsch and Belitski 2022) and with entrepreneurial education representing one of foundational conditions (GEM 2022).

It is important to bear in mind that the decisions leading to entrepreneurial activities are always based on individual motivations and deeper cognitive characteristics (Ferreira et al. 2019). Nevertheless, previous studies report a positive link between university-based entrepreneurship education and some entrepreneurial activities (Nabi et al. 2017). Their results stress the potential for exploring the impacts of other factors, specifically emotion-based and mindset related indicators with approaches to intention-to-behavior transitions exploring the contradictory findings returned by analysis of the contextual effects (considering person, context, and educational–models as moderators). In seeking to combat the multiple barriers, it is essential to overcome the conditions preventing recognition and/or the pursuit of opportunities (Khanin et al. 2022). Furthermore, it is equally crucial to conceive of entrepreneurship as a predictive-adaptive process with the balance relying on intuition and planning judgment (Rapp 2022).

As the work of Mukhtar et al. (2021) sets out, developing entrepreneurial mindsets might successfully accelerate the entrepreneurial intentions of university graduates. An entrepreneurial society deploys institutions designed and implemented to facilitate entrepreneurial activities as a driving force bringing about economic growth and prosperity (Audretsch 2014).

## Data and methods

This research applies the GEM 2017 Adult Population Survey (APS) Global National Data for Innovation-driven countries. The APS explores the role of individuals in entrepreneurial processes by focusing on business characteristics, the motivations of participants and entrepreneurial-related actions and attitudes. The 2017/18 GEM Global Report states that 54 economies around the world implemented the questionnaire. This generated information on entrepreneurial behavior in five regions - Europe, North America, Latin America, Caribbean, Africa, and Oceana - according to three economic development levels – Innovation-driven, Factor-driven, Efficiency-driven, merging 174,128 observations, randomly collected from the adult population aged between 18 and 64 years old. GEM APS makes available the TEA rate for measuring the prevailing entrepreneurial activities. The educational level allows for identifying the individuals with higher education degrees and enables analysis of the TEA of university graduates. We aim to investigate the relationship between the TEA of graduates (TEAed4) and variables exploring the entrepreneurial attitudes of these adults and the entrepreneurial social values prevailing towards entrepreneurial activities.

According to the GEM research community, there is general recognition that entrepreneurial attitudes and values return a positive effect on the TEA rate (Coduras et al. 2016). Thus, our study analyzes the capability to identify opportunities, obtain the skills and knowledge to start up a business, know someone who started a business in the last two years, the absence of fear of failure and perceptions of entrepreneurship as a good career choice, and associating entrepreneurship high social status, attention, and recognition.

Our methodological approach is quantitative – the regression method – and qualitative – the fsQCA method. To this end, we now present the two studies undertaken within a complementary perspective.

In the quantitative approach (Study 1), we attempt to recognize whether the attitudes and values towards entrepreneurship relate to TEAed4 with these statistical analyses carried out by IBM SPSS Statistics - Version 26. We performed multilinear regression analysis where the dependent variable is TEAed4. Two distinct models define the independent variables, attitudes – Model 1 – and values – Model 2.

In the qualitative approach (Study 2), we explore whether the attitudes and values towards entrepreneurship relate to TEAed4 through Fuzzy Set Qualitative Comparative Analysis (fsQCA), applying the attitudes – Model 1 – and values – Model 2 as causal conditions to achieve the outcome: TEAed4. The use of fsQCA has seen a significant rise in the fields of entrepreneurship and innovation research over the recent years (Kraus et al. 2018). The fsQCA fits within the widely used asymmetrical techniques today to more accurately predict and explain real-world business phenomena (Kumar et al. 2022).We adopted fsQCA software – version 3.0 – to apply the Fuzzy Set QCA method.

The complementary dimension of the approaches chosen interrelates with the need to complement the multilinear regression model results and explore the question of attitudes and social values, considering them as causal conditions to achieve the outcome of the levels of entrepreneurial activity performed by individuals holding graduate degrees.

Both studies incorporate the following models:

Model 1: *TEAed4* = f(*Knoent*, *Opport*, *Suskil*, *Frfail*).

Model 2: *TEAed4* = f(*Equali*, *NBgood*, *NBstat*, *NBmedi*).

Table 1 describes the independent and dependent variables applied in Study 1 and Study 2.

**Table 1:** Variable Description

		Variable	Description
Independent Variables (Study 1 + Study 2)	Model 1	Knowing Entrepreneurs (*Knoent*)	Applies the percentage of GEM 2017 APS survey respondents who responded positively to “*Knows someone who started a business in the past two years*”.
		Opportunities’ perception (*Opport*)	Applies the percentage of APS respondents who responded positively to “*Good conditions to start a business in the next six months in the area I live*”.
		Skills to start up (*Suskil*)	Applies the percentage of APS respondents who responded positively to “*Has required knowledge/skills to start a business*”.
		Fear of failure (*Frfail*)	Applies the percentage of APS respondents who responded positively to “*Fear of failure would prevent starting a business*”.
	Model 2	People prefer equal standards of living (*Equali*)	Applies the percentage of APS respondents who responded positively to “*People prefer equal standards of living for all*”.
		Entrepreneurship as a good career (*NBgood*)	Applies the percentage of APS respondents who responded positively to “*People consider starting a business as a good career choice*”.
		Entrepreneurship high social status (*NBstat*)	Applies the percentage of APS respondents who responded positively to “*People attach high status to successful entrepreneurs*”.
		Media spread entrepreneurship (*NBmedi*)	Applies the percentage of APS respondents who responded positively to “*In my country, there is lots of media attention for entrepreneurship*”.
Study 1 *Linear Regression* Dependent Variable		Innovation-driven TEA Graduate rate (TEAed4)	Applies the percentage of APS of adults with graduate experience who responded positively to “*Setting up firm or owner of a young firm*” – considering that they started or have been running a business for up to 3.5 years.
Study 2 *fsQCA Model* *Outcome*			

Source: Own elaboration.

## Overview of studies

### Study 1

**Fig. 2:**
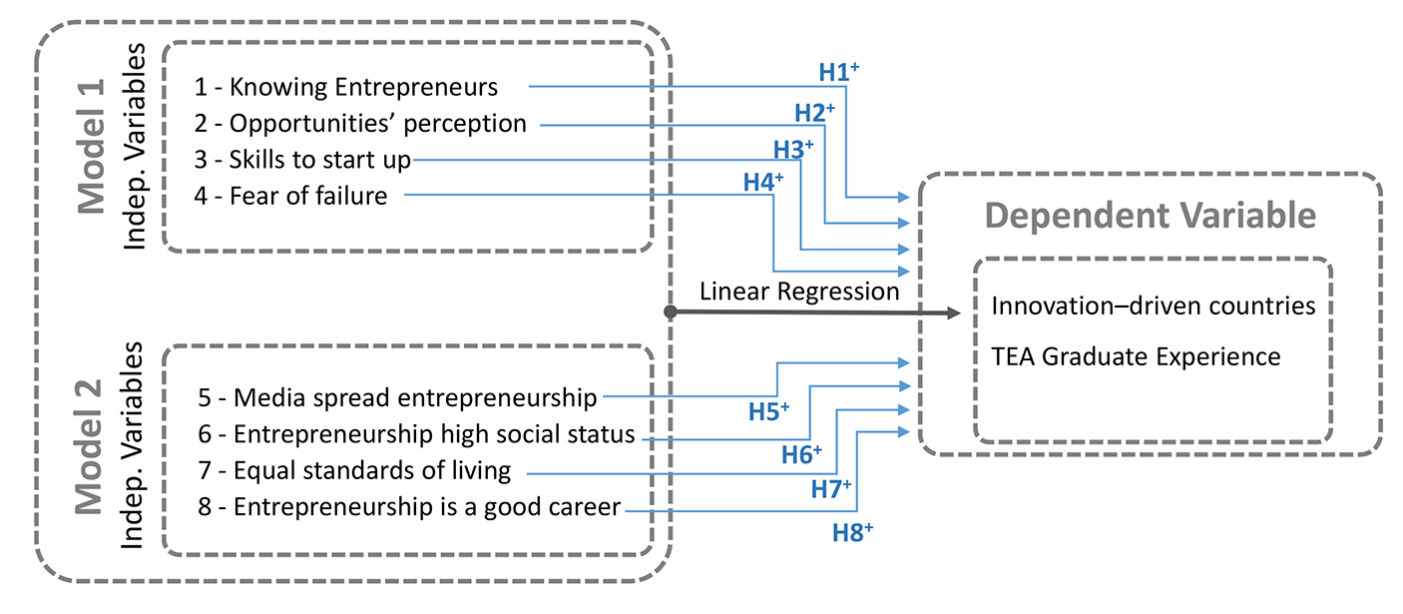
– Conceptual Model Source: Own elaboration.

Study 1 analyzes the statistical relationship between *TEAed4* and a set of variables reflecting the entrepreneurial attitudes of adults and the entrepreneurial social values prevailing. We carried out two multiple linear regressions using Model 1 and Model 2 variables based on the stepwise procedure. This study deploys a conceptual model (see Fig. 2) in which knowing recent entrepreneurs (*knoent*), perception of opportunities (*Opport*), skills to start up (*Suskil*), fear of failure (*Frfail*) – Model 1 – and the preference for equal standards of living (*Equali*), the perception that successful entrepreneurs get high social status (*NBstat*), consider entrepreneurship as a good career choice (*NBgood*) and the media properly covering national entrepreneurship (*NBmedi*) – Model 2 – positively influence forays into entrepreneurial activities undertaken by individuals with graduate degrees (*TEAed4*).

#### Model 1

*TEAed4 =* β_0_ + β_1_*Knoent* + β_2_*Opport* + β_3_*Suskil* + β_4_*Frfail* + ε.

#### Model 2

*TEAed4* = β_0_ + β_1_*Equali* + β_2_*NBstat* + β_3_*NBgood* + β_4_*NBmedi* + ε.

Table 2 presents a summary of the multiple linear regression analysis results.

**Table 2:** Stepwise multilinear regression models on the 2017 TEAed4 rate of innovation-driven countries

Dependent variable TEAed4 rate for innovation-driven countries participating in GEM							
Model 1	R square	32.4%	95% confidence	Model 2	R square	21.5%	95% confidence
	Independent variables	B	Significance		Independent variables	B	Significance
	Constant	-0.171	0.972		Constant	-0.890	0.895
	Skills to start up	0.313	0.009		Media spread entrep.	0.235	0.040
	Perception of opportunities	0.153	0.476		Entrep. high social status	-0.112	0.615
	Knowing entrepreneurs	-0.319	0.199		Equal standards of living	-0.088	0.729
	Fear of failure	-0.259	0.190		Entrep. good career	-0.107	0.621

Source: Own elaboration.

We then analyzed the assumptions of multilinear regression – normal distribution and homogeneity. We graphically validated the first two assumptions with VIF serving to diagnose multicollinearity and the efficiency of the model based on analysis of *R*^*2*^ and VIF. Model 1 attains significance and explains a relevant proportion of TEAed4 (*R*^*2*^ = 0.324) with Model 2 also significant and correspondingly explaining a relevant proportion of TEAed4 (*R*^*2*^ = 0.215).

The regression models identify the outcome as 32.4% (Model 1) and 21.5% (Model 2). Analysis of the entrepreneurial attitudes identifies the positive presence of the skills required to start a business as the most significant factor for achieving higher TEA rates. In studying entrepreneurial social values, positive media attention on entrepreneurship constitutes the most significant social value for generating high TEA rates. These results corroborate the results obtained by Coduras et al. (2016).

Hypotheses H1 (knowing recent entrepreneurs positively influences TEAed4), H2 (Perception of opportunities positively influences TEAed4), and H4 (Fear of failure positively influences TEAed4) did not gain support from Model 1. Furthermore, hypotheses H6 (Perception that successful entrepreneurs get high social status positively influences TEAed4), H7 (Considering entrepreneurship as a good career choice positively influences TEAed4), and H8 (Preference for equal standards of living positively influences TEAed4) did not obtain statistical significance in Model 2.

### Study 2

**Fig. 3:**
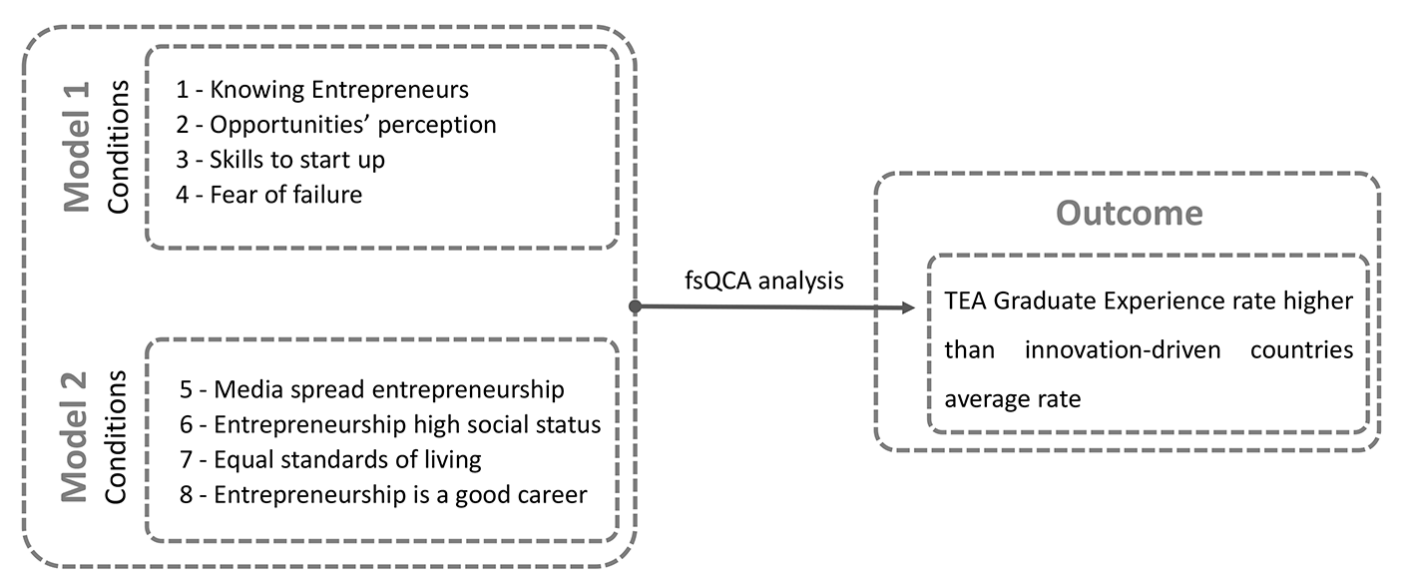
– Proposed Model Source: Own elaboration.

Study 2 aims to generate more detailed insights into explaining the relationship between *TEAed4* and the influences of entrepreneurial attitudes and social values over entrepreneurial activities, thus complementing the results of the regression models. This study applies fsQCA for which we performed a calibration in accordance with the direct method (Ragin 2007). The proposed model (Fig. 3) is based on Model 1 – conditions 1, 2, 3, and 4 and Model 2 – conditions 5, 6, 7, and 8. The variables included in each model depict the scores for the calibrated condition rates.

The direct method is based on three qualitative calibration anchors – the threshold for full non-membership, full membership, and the cross-over point (Ragin 2007).

The GEM innovation-driven countries reflect a country sample that varies each year according to the World Economic Forum classification. This classification represents different cultural backgrounds, transitions between efficiency-driven to innovation-driven and other specific factors that may condition the existence of extreme positive or negative values. Hence, this requires a foundation based on the recent trends in *TEAed4* (2008–2017) to justify the selection of the threshold (Coduras et al. 2016). Despite the availability of 2018 Global National APS Data, we undertaken analysis only until 2017. This decision derives from the change in the World Economic Forum classification, which now considers low income, middle income, and high income as a criterion in a classification that, following its application for the first time, would prevent the collection of the necessary past data to enable calibration. Table 3 presents the TEAed4 historical information.

**Table 3:** GEM TEAed4 historical information

TEAed4	2008	2009	2010	2011	2012	2013	2014	2015	2016	2017
Minimum values	0.11	5.64	0.33	5.16	2.55	3.11	4.70	4.59	3.23	3.64
Means	7.72	8.09	7.14	8.65	9.10	10.63	14.03	9.76	7.86	13.57
Maximum values	14.11	14.65	21.41	11.95	16.59	24.54	31.66	27.13	17.30	30.06

Source: Own elaboration.

The cross-over point conveys the value of the interval-scale variable that a particular case most belongs to – whether more in or out of the targeted outcome. According to the results, 10% establishes the cross-over point based upon the approximate *TEAed4* 10 year average.

Table 4 presents the original sample and the TEAed4 calibration – column “membership degree”. After completing this calibration, deploying a similar procedure to calibrate the Model 1 and Model 2 conditions, this study advanced with fsQCA analysis.

**Table 4:** Sample data and outcome of direct method calibration

Country	Knoent	Opport	Suskil	Frfail	Equali	NBgood	NBstat	NBmedi	TEAed4	COP	TEAed4-COP	Membership Degree
JP	18.88	7.41	10.77	43.96	43.01	24.27	51.96	56.17	3.64	10	-6.36	0.06
IT	20.47	28.78	30.39	51.11	68.92	64.17	73.21	54.94	6.43	10	-3.57	0.18
FR	33.19	34.13	36.31	38.3	53	59.06	74.21	47.04	7.61	10	-2.39	0.26
SE	36.11	79.49	34.5	43.07	64.31	53.6	70.52	64.69	7.82	10	-2.18	0.28
AE	65.96	35.45	64.79	53.57	82.33	82.73	87.77	84.46	8.24	10	-1.76	0.32
CY	37.96	51.01	46.35	54.85	51.93	66.2	61.53	50.51	9.19	10	-0.81	0.41
GR	22.8	13.74	43.4	70.19	61.26	63.36	66.49	43.35	10.05	10	0.05	0.50
QA	28.23	45.59	41.07	43.34	57.02	65.93	77.32	54.04	12.22	10	2.22	0.65
SW	33.86	47.16	42.09	35.23	60.23	53.02	73.2	58.96	12.43	10	2.43	0.66
NL	36.75	64.09	44.61	32.9	63	81	67.5	63.18	12.91	10	2.91	0.69
ES	32.96	31.86	44.8	43.55	71.39	53.83	47.88	50.91	14.17	10	4.17	0.76
IL	60.17	58.29	44.14	54.71	41.01	65.16	86.07	55.32	15.32	10	5.32	0.81
SI	38.98	34.6	53.31	34.77	80.45	55.12	73.42	72.65	15.54	10	5.54	0.82
LU	35.42	54.77	40.86	50.58	44.8	42.98	69.95	48.67	15.65	10	5.65	0.82
AU	34.84	51.39	49.3	41.79	79.03	53.87	68.91	74	15.69	10	5.69	0.83
KR	42.94	35.3	45.68	32.56	67.11	47.24	68.57	60.48	16.71	10	6.71	0.86
US	32.78	63.59	54.31	34.45	52.42	63.05	75.49	74.48	16.79	10	6.79	0.86
EE	46.49	60.95	49.72	36.84	57.01	54.22	64.74	60.99	18.27	10	8.27	0.91
PR	20.08	27.97	46.65	33.35	55.57	22.56	52.3	81.15	22.65	10	12.65	0.97
CA	39.21	60.23	55.59	47.17	74.85	65.61	73.96	76.5	30.06	10	20.06	1

Source: Own elaboration. *Knoent*: Knowing Entrepreneurs, *Opport*: Perception of opportunities, *Suskil*: Skills to start up, *Frfail*: Fear of failure, *Equali*: People prefer equal standards of living, *NBgood*: Entrepreneurship as a good career, *NBstat*: Entrepreneurship high social status, *NBmedi*: Media spread entrepreneurship, *TEAed4*: Innovation-driven TEA Graduate rate, *COP*: Cross over point.

Table 5 conveys how this condition is verifiable in all the solutions presented with levels of consistency attaining values between 0.750597 and 0.984542. Hence, and in keeping with the results, we may state that coverage of the conditions corresponds to at least 78% of the outcome.

**Table 5:** fsQCA Solutions for TEAedu4 according to Model 1 conditions

Model 1: TEAed4 = f(Knoent, Opport, Suskil, Frfail) Algorithm: Quine-McCluskey				
Frequency cutoff: 1 Consistency cutoff: 0.789934		Solution coverage: 0.811858		Solution consistency: 0.782774
		Raw coverage		Consistency
~Knoent*~Suskil		0.497233		0.750597
Knoent*Suskil		0.566798		0.892902
~Knoent*~Frfail		0.56917		0.862275
Suskil*~Frfail		0.654546		0.984542
Cases with greater than 0.5 membership in term ~ Knoent*~Suskil: Japan (0.95,0.06), Italy (0.78,0.18), France 0.62,0.26), Qatar (0.57,0.65), Switzerland (0.54,0.66), Luxembourg (0.53,0.82), Greece (0.51,0.5) Cases with greater than 0.5 membership in term Knoent*Suskil: United Arab Emirates (0.95,0.32), Estonia (0.69,0.91), Canada (0.58,1), Slovenia (0.57,0.82), Korea (0.56,0.86), Cyprus (0.55,0.41), Netherlands (0.52,0.69) Cases with greater than 0.5 membership in term ~ Knoent*~Frfail: Puerto Rico (0.94,0.97), USA (0.64,0.86), France (0.62,0.26), Switzerland (0.59,0.66), Australia (0.55,0.83), Qatar (0.54,0.65), Spain (0.53,0.76) Cases with greater than 0.5 membership in term Suskil*~Frfail: USA (0.81,0.86), Slovenia (0.79,0.82), Estonia (0.69,0.91), Australia (0.65,0.83), Puerto Rico (0.59,0.97), Korea (0.56,0.86), Spain (0.53,0.76), Netherlands (0.52,0.69)				

Source: Own elaboration.

Table 6 sets out the results demonstrating how this condition is verifiable in all the presented solutions with levels of consistency of between 0.780368 and 0.843949. Hence, and based on these results, we may state that condition coverage attains at least 75% of the outcome.

**Table 6:** fsQCA Solutions for TEAedu4 using Model 2 conditions

Model 2: TEAed4 = f(Equali, NBgood, NBstat, NBmedi) Algorithm: Quine-McCluskey				
Frequency cutoff: 1 Consistency cutoff: 0.821721		Solution coverage: 0.823715		Solution consistency: 0.746953
		Raw coverage		Consistency
~NBstat*~NBmedi		0.456126		0.792582
~Equali*~NBmedi		0.502767		0.780368
Equali*NBgood		0.490119		0.825566
NBgood*NBstat		0.56996		0.81377
~Equali*~NBgood*~NBstat		0.377075		0.816781
Equali*NBstat*NBmedi		0.418972		0.843949
Cases with greater than 0.5 membership in term ~ NBstat*~NBmedi: Spain (0.85,0.76), Cyprus (0.74,0.41), Japan (0.72,0.06), Greece (0.59,0.5), Estonia (0.54,0.91), Korea (0.52,0.86) Cases with greater than 0.5 membership in term ~ Equali*~NBmedi: Luxembourg (0.89,0.82), Cyprus (0.8,0.41), France (0.77,0.26), Israel (0.74,0.81), Japan (0.72,0.06), Qatar (0.64,0.65), Estonia (0.54,0.91), Switzerland (0.53,0.66) Cases with greater than 0.5 membership in term Equali*NBgood: United Arab Emirates (0.95,0.32), Canada (0.73,1), Italy (0.7,0.18), Netherlands (0.57,0.69), Greece (0.51,0.5) Cases with greater than 0.5 membership in term NBgood*NBstat: United Arab Emirates (0.95,0.32), Qatar (0.74,0.65), Israel (0.72,0.81), Canada (0.69,1), USA (0.67,0.86), Italy (0.66,0.18), France (0.56,0.26) Cases with greater than 0.5 membership in term ~ Equali*~NBgood*~NBstat: Japan (0.92,0.06), Puerto Rico (0.69,0.97), Estonia (0.56,0.91) Cases with greater than 0.5 membership in term Equali*NBstat*NBmedi: United Arab Emirates (0.95,0.32), Canada (0.69,1), Slovenia (0.67,0.82), Sweden (0.56,0.28)				

Source: Own elaboration.

Table 7 portrays the results of the necessary and sufficient conditions analysis of the TEAed4 outcome taking into account the Model 1 and Model 2 variables as conditions.

**Table 7:** Results for Necessary Conditions for Outcome – TEAed4

Model 1: *TEAed4* = f(*Knoent*, *Opport*, *Suskil*, *Frfail*)		
	**Consistency**	**Coverage**
*Knoent*	0.607115	0.862921
*~Knoent*	0.653755	0.745045
*Opport*	0.718577	0.885102
*~Opport*	0.557312	0.724563
*Suskil*	0.776285	0.913488
*~Suskil*	0.546245	0.747027
*Frfail*	0.452174	0.707921
*~Frfail*	0.766798	0.813758
Model 2: *TEAed4* = f(*Equali*, *NBgood*, *NBstat*, *NBmedi*)		
	**Consistency**	**Coverage**
*Equali*	0.646640	0.816367
*~Equali*	0.616601	0.781563
*NBgood*	0.648221	0.769953
*~NBgood*	0.622134	0.841711
*NBstat*	0.667194	0.800000
*~NBstat*	0.601581	0.805291
*NBmedi*	0.630830	0.864572
*~NBmedi*	0.598419	0.702878

Source: Own elaboration.

In Model 1, *~Frfail* constitutes a sufficient condition for a TEAed4 outcome with *Suskil* (0.913488), *Opport* (0.885102), and *Knoent* (0.862921) standing out as the most prominent necessary conditions. In Model 2, the most prominent necessary conditions are *NBmedi* (0.864572) and *~ NBgood* (0.841711).

Based on the complex solutions, fsQCA also allows for the presentation of possible paths for TEAed4 in accordance with the Model 1 and Model 2 conditions. Table 8 details the most relevant recipes returned by fsQCA and complementing the multilinear regression analysis presented in Study 1.

**Table 8:** Most relevant fsQCA recipes for *TEAed4*

Model 1: *TEAed4* = f(*Knoent*, *Opport*, *Suskil*, *Frfail*)	
Suskil*~Frfail	The possibility of adults with graduate degrees setting up a firm or owning a young firm is potentiated when individuals consider they hold the required knowledge/skills to start a business and report an absence of fear of failure that would prevent them from starting businesses. The countries with high membership scores for this outcome are USA, Slovenia, Estonia, Australia, Puerto Rico, Korea, Spain, and Netherlands.
Knoent*Suskil	The possibility of adults with graduate degrees setting up a firm or owning a young firm is potentiated when individuals consider they hold the required knowledge/skills to start businesses and know someone who started a business in the past two years. The countries with high membership scores for this recipe outcome are the United Arab Emirates, Estonia, Canada, Slovenia, Korea, Cyprus, and Netherlands.
Model 2: TEAed4 = f(*Equali*, *NBgood*, *NBstat*, *NBmedi*)	
Equali*NBgood	The possibility of adults with graduate degrees setting up a firm or owning a young firm is potentiated when individuals prefer the existence of equal standards of living for all and consider that starting a business is a good career choice. The countries with high membership scores for this outcome are the United Arab Emirates, Canada, Italy, Netherlands, and Greece.
Equali*NBstat*NBmedi	The possibility of adults with graduate degrees setting up a firm or owning a young firm is potentiated when individuals prefer the existence of equal standards of living for all, society attaches high status to successful entrepreneurs and the country devotes high media attention to entrepreneurship. The countries with high membership scores for this recipe outcome are the United Arab Emirates, Canada, Slovenia, and Sweden.

Source: Own elaboration.

The first recipe - Suskil*~Frfail – incorporates the presence of the knowledge/skills required to start businesses and the absence of fear of failure. The second recipe - Knoent*Suskil – also refers to the knowledge/skills required to start businesses alongside the importance of knowing someone who started a business in the past two years.

Considering the Model 2 conditions, the first recipe - Equali*NBgood – relays the importance of equal standards of living existing for all and the belief that starting a business constitutes a good career choice. The second - Equali*NBstat*NBmedi – incorporates the importance of equal standards of living for all alongside society attaching high status to successful entrepreneurs and the country devoting high levels of media attention to entrepreneurship.

## Discussion

The literature exploring the TEA rate of university graduates and variables depicting adult entrepreneurial attitudes and entrepreneurial social values have not yielded consistently strong results. Simultaneously, the level of university commitment to entrepreneurial training seems to return a discretely positive relationship considering the results obtained from the performed regression models. As a catalyst for regional economic and social development, entrepreneurial universities, within a Triple Helix context, enables a range of employability alternatives for their graduates while emphasizing the importance of launching new businesses for employment generation. Given this scenario, aligning university research and training priorities with the priorities of organizations represents an essential need.

According to the results, and despite not being generally accepted that entrepreneurial education provides a meaningful degree of preparedness for entrepreneurship, the regression models and fsQCA analysis convey how the likelihood of an adult with university experience setting up a firm or owning a young firm rises when such individuals consider they hold the knowledge/skills required for launching companies. Education and training configure the main factors for fostering entrepreneurship and reinforcing the need to endow graduate entrepreneurs with the capacity for developing sustainable entrepreneurial initiatives.

Therefore, whenever universities influence entrepreneurial activities in a particular country, they need to direct education processes towards entrepreneurship in order to foster entrepreneurial skills and capacities, thereby establishing entrepreneurial education and vocational training integrated into other fields of education. In this way, we may conclude that entrepreneurship can be taught and that universities represent the right environment for this. Furthermore, both the regression models and the fsQCA analysis also stress the importance of media outlets paying prominent attention to entrepreneurship, contributing to constructing an emotion-based and entrepreneurial mindset and successfully nurturing the acceleration of university graduates entrepreneurial intentions and building an entrepreneurial culture that supports economic growth and prosperity.

The articulation of quantitative and qualitative methodologies opens a range of promising possibilities through providing methodological synergies that expand the results from quantitative analysis - which are the most significant relevant factors for later entrepreneurial activities – by combining them with the qualitative results that return insights into the combinations of the factors analyzed and also characterizing the context where they occurs. Hence, the combination of Study 1 and Study 2 generates more extensive answers than those returned by the results of the regression models, deepening the information explaining entrepreneurial activity levels of graduates.

The results of Study 1 lend support to the hypotheses H3 (Start up skills positively influence TEAed4) - Model 1 and H5 (Appropriate media coverage of national entrepreneurship positively influences TEAed4) - Model 2 as a positive influence on TEAed4. Adults with graduate degrees and entrepreneurial skills and who attach importance to the attention paid by the media to entrepreneurship are therefore more likely to engage in entrepreneurial activities. These results confirm those obtained by Coduras et al. (2016) and correspondingly reinforce the importance of entrepreneurship education in developing the skills necessary to pursue entrepreneurial behaviors (Ruiz et al. 2020), within the scope of which universities may supply not only technical skills but also provide infrastructures (Bedő et al. 2020). These results also highlight the importance of wider media coverage, which enhances visibility of entrepreneurial impacts on the economy and that enable the identification of entrepreneurship as a highly desirable path (Muralidharan and Pathak 2017). Within this framework, Model 1 underpins the affirmation that knowing recent entrepreneurs, the perception of opportunities and the absence of fear of failure do not significantly relate to undertaking entrepreneurial activities.

Furthermore, Model 2 identifies how successful entrepreneurs receiving high social status, perceptions of entrepreneurship as a good career choice and the preference for equal standards of living do not significantly relate to engaging in entrepreneurial activities. We may thus conclude that the graduate TEA rate in innovation-driven countries can benefit from actively developing entrepreneurial skills and the media devoting a high level of attention to positive entrepreneurship related information.

Identification of the various types of entrepreneurship present in GEM data allows for the fsQCA approach to return a conceptual framework that enriches our understanding of the contributions each of the different conditions makes to TEAed4 outcomes.

Based on Study 2 fsQCA analysis, the results of the most prominent necessary conditions are *Suskil* (0.913488), *Opport* (0.885102), and *Knoent* (0.862921) – in Model 1 – and *NBmedi* (0.864572) and *~ NBgood* (0.841711) – in Model 2 - confirming and enabling the extension of the results made possible by the regression models. Analysis of the most relevant fsQCA recipes for TEAed4 in accordance with the Model 1 conditions allows for reflection on pertinent points potentially justifying these solutions in accordance with the groups of countries they occur in. The first recipe - Suskil*~Frfail – refers to the presence of the knowledge/skills required to start a business and the absence of fear of failure. This recipe becomes viable in the USA, Slovenia, Estonia, Australia, Puerto Rico, Korea, Spain and the Netherlands, countries with significant entrepreneurial cultures, high levels of development, increased commitments to entrepreneurial education and access to modern financial channels. This reinforces a positive link between university-based entrepreneurship skills and some entrepreneurial activities (Nabi et al. 2017) and the paramount importance of exploring the mindsets prevailing among entrepreneurial university graduates as regards their entrepreneurial intentions (Mukhtar et al. 2021).

The second recipe - Knoent*Suskil – also refers to the knowledge/skills required to start a business and the importance of knowing someone who started a business in the past two years. This recipe is viable for United Arab Emirates, Estonia, Canada, Slovenia, Korea, Cyprus and the Netherlands. The literature suggests that when individuals knowing someone personally who started a business in the past two years (Davidsson and Honig 2003) generates a second-hand experience of entrepreneurship that emerges as a relevant driver of entrepreneurial intentions through learning from the experiences, feelings or actions of another (Davidsson 1991).

Considering the Model 2 conditions, the first recipe - Equali*NBgood – relays the importance of equal standards of living existing for all and the belief that starting a business is a good career choice. This recipe is viable in the United Arab Emirates, Canada, Italy, the Netherlands and Greece. The second recipe - Equali*NBstat*NBmedi – encapsulates the importance of equal standards of living for all, people attaching high status to successful entrepreneurs and the country devoting significant media attention to entrepreneurship. The importance of entrepreneurship is also equality based as entrepreneurs often focus on solving problems, finding solutions to many issues that would otherwise be hard or nearly impossible to solve and, while benefiting themselves, also benefiting the rest of society (Hernández-Sánchez et al. 2019).

Theoretical implications.

This research provides substantial theoretical contributions to the literature. Considering the relevance of the entrepreneurial process outputs - new job and value creation, and their respective outcomes - socio-economic development, exploring all research strands is essential to generate a basis for actions in support of developing entrepreneurship. Firstly, the findings demonstrate that articulating quantitative and qualitative methodologies enables an expansion of the results returned by the quantitative analysis, unveiling combinations among the factors analyzed and exploring the context in which they occur. Secondly, this study strengthens the appropriateness of the Institutional and Planned Behavior theoretical frameworks and not only emphasising the importance of institutional or organizational factors as determinants of entrepreneurship (Abreu et al. 2016) but also the need to focus on the linkage between entrepreneurial attitudes, entrepreneurial intentions, and entrepreneurial behavior (Ajzen 1991). There is thus now recognition of the relationship between university support for entrepreneurship and the development of entrepreneurial activities (Coduras et al. 2008) based on the role of entrepreneurial education and approaching this as the driver of successful entrepreneurial behavior (Fayolle et al. 2006). In this scenario, entrepreneurial universities play a preponderant role as aggregators of capabilities (Audretsch et al. 2019) and the subsequent linkage between innovation and the entrepreneurial ecosystem (Autio et al. 2014) in a relational system that should function from a mutualist perspective (Schaeffer et al. 2021).

The study also very much highlights the importance of positive media attention to entrepreneurship and the most significant social value as regards achieving high TEA rates. We were furthermore able to conclude that adults with graduate degrees and entrepreneurial skills attach importance to this attention paid by the media to entrepreneurship and correspondingly display a greater likelihood to engage in entrepreneurial activities. In this context, exploring the media’s approaches to entrepreneurship represents an essential research objective. This crucially involves conveying the success and failure stories associated with entrepreneurial activities and raising general awareness of the scope for actions and reactions to each different situation.

In addition, establishing specific recipes with different combinations of conditions and their association with specify countries where those recipes hold validity enables the definition of more targeted and effective policies in keeping with the economic, social, and historical particularities framing each country’s particular situation at any given moment, scenarios that require theoretical exploration for later, duly contextualized, application.

Practical Implications.

There is now general recognition that universities constitute one of the core pillars of any entrepreneurial ecosystem (Guerrero et al. 2017). The emergence of entrepreneurial universities, as catalysts for regional economic and social development (Etzkowitz et al. 2000; Abreu et al. 2016), comprises the development of mindsets that equates to the existence of market opportunities.

The Triple Helix concept describes the essential need to align university research and training priorities with the goals of organizations in the region, enabling a range of employment alternatives for their graduates – whether self-employed, academic entrepreneurs, or intrapreneurs. Additionally, taking into account the impacts of launching new businesses and companies on the creation of employment, with education and training standing out as the main factors for fostering entrepreneurship, reinforces the need to endow graduate entrepreneurs with the capacity to develop sustainable entrepreneurial initiatives (Coduras et al. 2008).

Although attention mostly focuses on the skills needed for further entrepreneurial activities, we also need to explore the media attention to entrepreneurship. Given that positive media attention to entrepreneurship accounts for the most significant social value for achieving high TEA rates, we need to work on how we communicate entrepreneurial cases outputs and achievements.

Despite the setbacks in determining which factors broadly influence entrepreneurial performance and recognition of universities as the means of impacting on entrepreneurial attitudes and values, we need to gather out efforts around all the opportunities to bring about this development. Based on that detailed above, advancing with development of the fsQCA models is crucial to establishing the different combinations in the multiple and interrelated entrepreneurial conditions able to underpin effective and sustainable public policies most appropriate to the characteristics of each country.

Limitations and Future Research.

Our study holds limitations that further investigations may overcome. First, our analysis only applies to GEM TEAed4 data on innovation-driven countries. Possible lines of future research might deploy this methodology to analyze other educational levels, accessible through TEAed1, TEAed2, and TEAed3 variables, which captures the percentage of APS of adults with lower secondary, secondary or post-secondary education qualifications respectively. Another possibility derives from the need to weight this analysis as entrepreneurial activities may arise out of need or opportunity. Secondly, the need to work on the way to measure the performance of entrepreneurial universities remains outstanding. Despite the existence of instruments that allow for institutional self-analysis, such as HEInnovate, developed in a joint effort by the Organisation for Economic Cooperation and Development and the European Community, there remains the need to ascertain the tools to evaluate universities and their performance in accordance with entrepreneurship. This type of evaluation might return a means of directing university graduates with more entrepreneurial aptitudes towards those universities more qualified for teaching and providing future support for entrepreneurial activities. Thirdly, and according to the 2021/2022 GEM Global Report (GEM 2022), recognizing the paramount importance of entrepreneurship as a critical driver of economic development and recovery, there is also the need to understand the impacts on entrepreneurship driven by the COVID-19 pandemic. These questions arise because entrepreneurship can solve countless economic, environmental and social challenges. While positive media attention may significantly impact on entrepreneurial activities, exploring utilisation of all digital infrastructures reflects another core research goal. The pandemic has highlighted the importance of digital ecosystems as ‘invisible infrastructures’ that encourages emerging entrepreneurs (Fernandes et al. 2022). In the digital context, where metaverse technology appears as a future center of gravity for online social interactions (Kraus et al. 2022), it is crucial to approach just how entrepreneurs can prepare to deal with this new reality and leverage all of its opportunities.

## Data Availability

The data on GEM 2017 Adult Population Survey (APS) Global National Data for Innovation-driven countries is publicly available from the webpages of the GEM global Report.
